# Fresnel Prism on Hess Screen Test

**DOI:** 10.1155/2013/187459

**Published:** 2013-04-23

**Authors:** Kyung Min Koh, Ungsoo Samuel Kim

**Affiliations:** ^1^Department of Ophthalmology, Kim's Eye Hospital, Youngdeungpo 4th 156, Youngdeungpo-gu, Seoul 150-034, Republic of Korea; ^2^Department of Ophthalmology, Konyang University College of Medicine, Daejeon 302-832, Republic of Korea

## Abstract

A 65-year-old male patient complained of diplopia after a cataract surgery. He had esotropia of 18 prism diopters (PDs) at distant and near deviation, and therefore, we performed the Hess screen test to identify any abnormal eye movement. However, the indicator was found to be out of bounds, and therefore, the test could not be completed. Therefore, the test was subsequently performed with a 20 PD base-out Fresnel prism, and an abduction deficit was observed in the right eye, but not in the left eye. Therefore, we speculated that the patient had abducens nerve palsy in the right eye. The results obtained in the present study imply that performing the Hess screen test with the Fresnel prism may be very useful in diagnosing ambiguous abnormalities in patients with extraocular movement. The Hess screen test can be performed for patients with a strabismus of greater than 15 PD by using a Fresnel prism. Thus, a Fresnel prism may be useful for performing both the Hess screen test and Lancaster screen test.

## 1. Introduction

The Hess screen test is used for diagnosing ocular motility disorders in patients with normal sensory status. During the Hess screen test, the subject views a sequence of red targets on a tangent screen (placed at a distance of 50 cm) with 1 eye through a red filter and simultaneously views the targets with the other eye through a green filter, while directing a green laser spot such that it apparently superimposes the red targets. Thus, the test evaluates isolated movements of each eye by dissociating binocular vision with red and green filters [1]. This procedure allows one to distinguish between concomitant and incomitant strabismus such as paralytic strabismus. In the case of paralytic strabismus, the paretic muscle can be identified easily [2]. 

However, performing the Hess screen test is not possible in patients with a large-angle strabismus because the indicator falls out of bounds. Therefore, in the case of our patient, we performed the Hess screen test using a Fresnel prism (3 M Health Care, St. Paul, MN, USA), which is made of polyvinyl chloride and increases chromatic dispersion and reduces contrast [3].

## 2. Case Report

**Figure 1:**
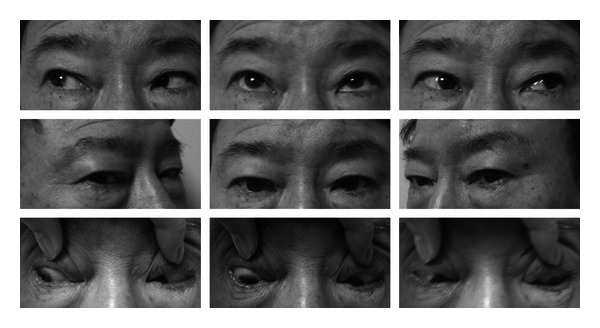
A 9-gaze photography shows esodeviation. No abduction deficit is noted.

The patient was a 65-year-old man who underwent phacoemulsification with intraocular lens implantation surgery in his left eye on September 17, 2011. Since then, he complained of diplopia and limited adduction in his right eye. He underwent an alternate prism cover test at our hospital on December 7, 2011; the results of the test showed that the patient had esotropia of 18 prism diopters (PDs) at distant and near deviation without correction. The alternate prism cover test that was performed also showed esotropia of 18 PDs at distant deviation without correction, and 9-gaze photography was performed on the same day (Figure 1).

**Figure 2:**
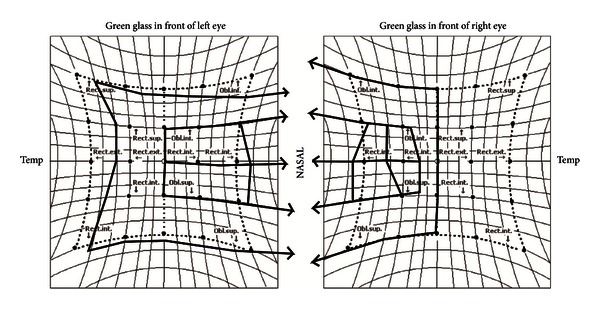
After the 20 PD base-out Fresnel prism, the abduction deficit is noted dominantly in the right eye.

The Hess screen test performed on December 22, 2011 showed that the indicator was out of bounds, and therefore, the test could not be completed. Therefore, the test was performed using a 20 PD base-out Fresnel prism; an abduction deficit was observed in the right eye, but not in the left eye (Figure 2). Therefore, we speculated that the patient had abducens nerve palsy in the right eye. 

## 3. Discussion 

Abducens nerve palsy presents as a horizontal diplopia, and diplopia is usually worse at distance and in lateral gaze of the paretic muscle. Several tests including duction/version test, forced duction test, Hess screen test, magnetic resonance imaging test, and serologic tests are helpful to differentiate the causes of the abducens nerve palsy [4].

The results of the present study imply that performing the Hess screen test with a Fresnel prism may be very useful in diagnosing ambiguous abnormalities in patients with extraocular movement. Although Tangent screen is useful in the patient with more than 15° and the distance from patient to Hess screen also can be adjusted, the Hess screen test can be performed in patients with a strabismus of greater than 15 PD by using a Fresnel prism without any change of test distance. Therefore, the Fresnel prism may be useful for performing both the Hess screen test and Lancaster screen test if Tangent screen is not available or there is not enough distance to perform test.
